# Entropy Generation Analysis and Thermodynamic Optimization of Jet Impingement Cooling Using Large Eddy Simulation

**DOI:** 10.3390/e21020129

**Published:** 2019-01-30

**Authors:** Florian Ries, Yongxiang Li, Kaushal Nishad, Johannes Janicka, Amsini Sadiki

**Affiliations:** 1 Institute of Energy and Power Plant Technology, Technische Universität Darmstadt, 64287 Darmstadt, Germany; 2 Laboratoire de Modelisation Mecanique, Energetique et Materiaux, Institut Superieur des Techniques Appliquees, B.P. 6534 Kinshasa 31 NDOLO, D.R. Congo

**Keywords:** entropy generation analysis, large eddy simulation, turbulent flows, heat transport, jet impingement cooling

## Abstract

In this work, entropy generation analysis is applied to characterize and optimize a turbulent impinging jet on a heated solid surface. In particular, the influence of plate inclinations and Reynolds numbers on the turbulent heat and fluid flow properties and its impact on the thermodynamic performance of such flow arrangements are numerically investigated. For this purpose, novel model equations are derived in the frame of Large Eddy Simulation (LES) that allows calculation of local entropy generation rates in a post-processing phase including the effect of unresolved subgrid-scale irreversibilities. From this LES-based study, distinctive features of heat and flow dynamics of the impinging fluid are detected and optimal operating designs for jet impingement cooling are identified. It turned out that (1) the location of the stagnation point and that of the maximal Nusselt number differ in the case of plate inclination; (2) predominantly the impinged wall acts as a strong source of irreversibility; and (3) a flow arrangement with a jet impinging normally on the heated surface allows the most efficient use of energy which is associated with lowest exergy lost. Furthermore, it is found that increasing the Reynolds number intensifies the heat transfer and upgrades the second law efficiency of such thermal systems. Thereby, the thermal efficiency enhancement can overwhelm the frictional exergy loss.

## 1. Introduction

Jet impingement cooling has been widely used as means of heat transfer equipment in a variety of engineering applications such as cooling of gas turbine blades or electronic components. It provides a very effective and flexible way to transfer thermal energy and enables up to three-fold higher heat transfer coefficients compared to conventional flow arrangements like fully developed pipe or channel flows [1]. Thereby, the performance of jet impingement cooling depends on a large number of operating parameters (e.g., jet-to-plate spacing, target plate inclination, Reynolds number) and it features very complex heat and fluid flow dynamics (e.g., stagnation points, shear flow boundary layers, anomalies in the distribution of the Nusselt number). It is, therefore, not surprising that in the last few decades, jet impingement cooling has been the subject of extensive research to gain insights into the complex physics of impinging flows and to identify favorable operating conditions along with guidelines for their practical usage. An overview of experiments, numerical investigations and empirical correlations can be found in [1,2,3,4,5,6,7,8] and elsewhere.

Despite the significant progress towards a better understanding of the physics and applicability of jet impingement cooling, many issues remain still open. In particular, the large number of operating parameters, complex heat and fluid flow dynamics and turbulent flow conditions (typically in the range of Re = 4000–80,000 [1]) impede a complete description of jet impingement cooling and make the optimization of such thermal devices very difficult. In this respect, it was shown by Ries et al. [9] that an analysis based on entropy generation is a promising approach to provide not only a deeper insight into the complex physical mechanisms of impinging flows but also to localize possible irreversibilities within such thermal devices. Thermodynamic irreversibility in thermo-fluid systems manifests itself as a loss of degree of freedom in the description of the material behavior, as well as the turbulence structure of the flow in the fluid [10]. There are many causes of irreversibilites such as mechanical dissipation, heat conduction, diffusion, chemical reactions, or Joule heating. Based on the second law of thermodynamics, such irreversibilities cause a degradation of available energy into internal energy in the working fluid leading to an increase of the system entropy. This increase in the entropy of a thermal system is called entropy generation [11] which leads to a reduction of the thermodynamic efficiency of a system [12]. Entropy generation analysis is based on the second law of thermodynamics in conjunction with heat transfer and fluid mechanics principles and it allows evaluation of the significance of irreversibilities related to heat transport and friction in thermo-fluid systems. From an engineering perspective, the concept of entropy generation analysis can be therefore useful in order to characterize the evolution of thermal processes and also as a design tool in order to avoid the imminent loss of available mechanical power in thermo-fluid systems [13,14], likewise for the conceptual design of jet impingement cooling applications. Various investigations on entropy generation have been reported in the literature for different configurations and physical processes using numerical and analytical approaches, e.g., [11,15,16,17,18,19,20,21,22]. A detailed description of the theoretical background of entropy generation analysis can be found in [23,24] and elsewhere.

In dealing with entropy generation analysis using computational fluid dynamics (CFD), usually the local form of the second law of thermodynamics is used to investigate thermodynamic irreversibilities. This allows quantification of the overall entropy generation of a system and to examine how irreversibilities are distributed locally throughout the system [24]. Based on the concept of minimal entropy generation and CFD, causes of irreversibilities have been analyzed for a wide range of thermo-fluid processes including laminar and turbulent heat transfer in wall-bounded flows [25,26,27,28,29,30,31,32,33], flows under supercritical thermodynamic conditions [34,35,36,37], reacting flows [38,39,40,41] and also in heat transfer in impinging flows [9,42,43]. Contributions of the theory and application of entropy generation analysis using CFD for different types of engineering systems are reviewed in [24,44,45].

Focusing on entropy generation analysis of turbulent heat and fluid flows using CFD, only a few direct numerical simulations (DNS) are reported in the literature (e.g., [9,34,35,41,46,47]). All these DNS studies are restricted to heat and fluid flow problems at low-to-moderate Reynolds numbers and simple geometries due to the high computational cost of DNS. In contrast, entropy generation analysis based on the solution of the Reynolds-averaged Navier-Stokes equations (RANS) have been carried out in many numerical studies (e.g., [26,27,29,30,31,33,36,37,39,42]) because of the relatively low computational cost of the RANS approach. However, it is well known that the prediction of complex heat and fluid flows based on RANS is not always accurate for many cases, especially for turbulent flows with large scale, unsteady characteristics. To overcome the limitations of DNS and RANS, many researchers paid more attention towards large eddy simulations (LES) as an alternative method to predict turbulent heat and fluid flows. In LES, large three-dimensional unsteady turbulent motions are explicitly computed, while a turbulence closure model accounts for the influence of the unresolved more universal small scales. This simplifies the turbulence modeling, reduces the computational effort compared to DNS and improves the predictive capability in comparison to RANS. Despite the great potential of LES, entropy generation analysis based on LES are rarely reported in the literature. This is largely because of the difficulties in modeling of the unresolved subgrid-scale entropy production rates. Recently, Safari et al. [40,48] developed a methodology based on filtered density function (FDF) approach that allows accurate LES predictions of the unresolved entropy generation in turbulent reacting flows. However, the proposed FDF approach requires the solution of an additional transport equation for the entropy filtered density function and can be therefore not used as a simple post-processing tool, likewise in a commercial CFD code. Other entropy generation analyses based on LES that includes the subgrid-scale contribution of the entropy production are not reported in the literature.

In this paper, we present novel model equations that allow calculation of local entropy generation rates in the post-processing phase of LES for turbulent heat and fluid flows. Thereby, the effects of subgrid-scale entropy production are taken into account by means of algebraic equations based on the resolved turbulent quantities without solving additional transport equations. Thus, the presented approach can be used as a simple post-processing tool, does not require much computational effort, can be applied with common subgrid-scale eddy viscosity models, and remains easy to implement into existing academic and commercial CFD codes. The proposed LES approach is then employed to evaluate and optimize the thermodynamic performance of a turbulent jet impinging on a heated surface based on second law analysis. In particular, the influence of plate inclination and Reynolds number on the thermal performance of such flow arrangements is investigated. In this respect, distinctive features of heat and flow dynamics of the impinging fluid are detected and optimal operating designs for such thermal devices identified.

The paper is organized as follows. Section 2 outlines the novel approach using the entropy generation analysis within the LES framework including the criteria to judge the thermodynamic performance of jet impingement cooling devices. Next, the configuration under investigation, a turbulent impinging jet on a heated plate, is shortly introduced in Section 3. Subsequently, the achieved results of the LES study are presented and discussed in Section 4. In particular, irreversibilities in jet impingement cooling are analyzed and optimal designs for such thermal devices are identified. Finally, some concluding remarks are summarized in Section 5. A detailed evaluation of the proposed, novel model equations to calculate local entropy generation rates using LES are provided in the Appendix C for the sake of completeness.

## 2. Modeling

At first the LES framework to calculate turbulent fluid flow with convective heat transport is presented in this section. Then, the entropy generation analysis based on LES along with the criteria to judge the thermodynamic performance of impingement cooling devices are presented. Notice that the applied numerical approach was already verified and validated by the authors in previous studies (see [9,49]). A detailed code validation and verification is therefore not included in this work.

### 2.1. LES Framework

Large eddy simulation of turbulent heat transport with constant physical properties is carried out. Thereby, the temperature is treated as a passive scalar and buoyancy effects are neglected. Based on these assumptions, the LES balance equations of continuity, momentum and energy read:

(1) $$\frac{\partial {\bar{U}}_{i}}{\partial x_{i}}=0,$$

(2) $$\frac{\partial {\bar{U}}_{i}}{\partial t}=-\frac{\partial }{\partial x_{j}}\left({\bar{U}}_{i}{\bar{U}}_{j}\right)-\frac{\partial \bar{P}}{\partial x_{i}}+\frac{\partial }{\partial x_{j}}\left(\nu \left(\frac{\partial \bar{U_{i}}}{\partial x_{j}}+\frac{\partial \bar{U_{j}}}{\partial x_{i}}\right)\right)-\frac{\partial \tau_{\langle ij\rangle }^{sgs}}{\partial x_{j}},$$

(3) $$\frac{\partial \bar{T}}{\partial t}=-\frac{\partial }{\partial x_{j}}\left({\bar{U}}_{j}\bar{T}\right)+\frac{\partial }{\partial x_{i}}\left(\frac{\nu }{Pr}\frac{\partial \bar{T}}{\partial x_{i}}\right)-\frac{\partial q_{i}^{sgs}}{\partial x_{i}},$$

where ${\bar{U}}_{i}$ is the velocity, $\bar{T}$ the temperature, ν the molecular viscosity, Pr the molecular Prandtl number, $\tau_{\langle ij\rangle }^{sgs}$ the deviatoric part of the subgrid-scale stress tensor, $q_{i}^{sgs}$ the subgrid-scale heat flux vector and *P* is the modified kinematic pressure, which includes the isotropic part of the subgrid-scale tensor. Filtered variables are represented by $\bar{\left(.\right)}$, while ${\left(.\right)}^{sgs}$ denotes subgrid-scale quantities. Notice that the viscous dissipation and pressure dilatation terms in the energy equation (Equation (3)) are usually assumed to be small in incompressible flows [50], and are therefore neglected in the present study.

To close the LES balance equations (Equations (1)–(3)), the eddy viscosity and eddy diffusivity approaches are employed in this work. These lead to the following formulations for the deviatoric part of the subgrid-scale stress tensor

(4) $$\tau_{\langle ij\rangle }^{sgs}=\nu_{sgs}\left(\frac{\partial \bar{U_{i}}}{\partial x_{j}}+\frac{\partial \bar{U_{j}}}{\partial x_{i}}\right)$$

and the subgrid-scale heat flux vector

(5) $$q_{i}^{sgs}=\alpha_{sgs}\frac{\partial \bar{T}}{\partial x_{i}}.$$

Here, $\nu_{sgs}$ is the subgrid-scale eddy viscosity, which is modeled in the present work using the wall-adapted linear eddy viscosity model (WALE) with the standard model coefficient of $C_{W}=0.5$ [51]. $\alpha_{sgs}$ is the subgrid-scale heat diffusivity, which is represented based on the Reynolds analogy $\alpha_{sgs}=\nu_{sgs}/Pr_{sgs}$, where the subgrid-scale turbulent Prandtl number is selected as $Pr_{sgs}=1$.

The LES balance equations (Equations (1)–(3)) are numerically solved using a projection method proposed in [52] combined with a three-stage explicit Runge-Kutta scheme of second-order accuracy for time integration [53], which were added to the open source C++ library OpenFOAM 2.4.0. Thereby, a second-order flux-limited differencing scheme is used for the convection terms and a conservative scheme is applied for the Laplacian and gradient terms. A detailed description, verification and validation of the code can be found in [9,49].

### 2.2. Entropy Generation Analysis Using LES

Since the entropy generation is directly linked to the dissipation of energy which is predominantly a subgrid-scale process in the context of LES, appropriate closures are of utmost importance once dealing with the entropy balance equation in which flow and heat transport processes contribute to the entropy production. In the case of non-reacting, single phase, and single component fluid flow with Fourier heat conduction, the filtered second law of thermodynamics can be expressed in the form of the filtered imbalance of entropy given as

(6) $$\frac{\partial \bar{\rho }\bar{s}}{\partial t}+\frac{\partial }{\partial x_{j}}\left(\bar{\rho }\bar{U_{j}s}\right)+\frac{\partial }{\partial x_{j}}\bar{\left(\frac{q_{j}}{T}\right)}={\bar{\Pi }}_{v}+{\bar{\Pi }}_{q}\geq 0,$$

where the terms on the left-hand side denote the local change, the convection and the flux of entropy density $\bar{s}$ (from left to right). The last two terms on the right-hand side represent the filtered entropy production rate by viscous dissipation ${\bar{\Pi }}_{v}$ and the filtered entropy production rate by heat transport ${\bar{\Pi }}_{q}$. Both terms, ${\bar{\Pi }}_{v}$ and ${\bar{\Pi }}_{q}$, are responsible for irreversibilities evolving in thermo-viscous fluid flow and need to be calculated in the case of entropy generation analysis. In the case of a Navier-Stokes-Fourier fluid flow with constant physical properties, these terms can be formulated in the context of LES as

(7) $${\bar{\Pi }}_{v}=\bar{\frac{1}{T}\tau_{ij}\frac{\partial U_{i}}{\partial x_{j}}}=\bar{\frac{\rho \nu }{T}\left(\frac{\partial U_{i}}{\partial x_{j}}+\frac{\partial U_{j}}{\partial x_{i}}\right)\frac{\partial U_{i}}{\partial x_{j}}},$$

(8) $${\bar{\Pi }}_{q}=\bar{\frac{1}{T^{2}}q_{j}\frac{\partial T}{\partial x_{j}}}=\bar{\frac{\lambda }{T^{2}}\frac{\partial T}{\partial x_{j}}\frac{\partial T}{\partial x_{j}}},$$

which are unclosed terms that cannot be directly calculated by the resolved velocity and temperature fields.

However, assuming that the mean of the filtered entropy production rates by viscous dissipation $\langle {\bar{\Pi }}_{v}\rangle$ is approximately the same as the mean of unfiltered entropy production rate $\langle \Pi_{v}\rangle$ leads to

(9) $$\langle \Pi_{v}\rangle \approx \langle {\bar{\Pi }}_{v}\rangle =\underset{\langle \Pi_{v}^{res}\rangle }{\underbrace{\langle \frac{\bar{\rho }\;\bar{\nu }}{\bar{T}}\left(\frac{\partial {\bar{U}}_{i}}{\partial x_{j}}+\frac{\partial {\bar{U}}_{j}}{\partial x_{i}}\right)\frac{\partial {\bar{U}}_{i}}{\partial x_{j}}\rangle }}+\underset{\langle \Pi_{v}^{sgs}\rangle }{\underbrace{\left(\langle {\bar{\Pi }}_{v}\rangle -\langle \frac{\bar{\rho }\;\bar{\nu }}{\bar{T}}\left(\frac{\partial {\bar{U}}_{i}}{\partial x_{j}}+\frac{\partial {\bar{U}}_{j}}{\partial x_{i}}\right)\frac{\partial {\bar{U}}_{i}}{\partial x_{j}}\rangle \right)}}$$

and similar for the entropy production rate by heat transport

(10) $$\langle {\bar{\Pi }}_{q}\rangle \approx \langle \Pi_{q}\rangle =\underset{\langle \Pi_{q}^{res}\rangle }{\underbrace{\langle \frac{\bar{\lambda }}{{\bar{T}}^{2}}\frac{\partial \bar{T}}{\partial x_{j}}\frac{\partial \bar{T}}{\partial x_{j}}\rangle }}+\underset{\langle \Pi_{q}^{sgs}\rangle }{\underbrace{\left(\langle {\bar{\pi }}_{\theta }\rangle -\langle \frac{\bar{\lambda }}{{\bar{T}}^{2}}\frac{\partial \bar{T}}{\partial x_{j}}\frac{\partial \bar{T}}{\partial x_{j}}\rangle \right)}},$$

where $\langle .\rangle$ denotes temporal or spatial averaging. To close Equations (9) and (10) the averaged unresolved terms of the entropy production, $\langle \Pi_{v}^{sgs}\rangle$ and $\langle \Pi_{q}^{sgs}\rangle$, have to be modeled, while the resolved entropy production terms, $\langle \Pi_{v}^{res}\rangle$ and $\langle \Pi_{q}^{res}\rangle$, can be directly calculated from the resolved velocity and temperature fields. Physically, Equations (9) and (10) imply that mechanical and thermal entropy generation can be split into a contribution of both large scales and smaller subgrid-scale structures, respectively. In particular, the first part includes entropy generation by mean and resolved quantities, while the subgrid-scale part additionally takes into account the remaining contribution of unresolved fluctuations.

By the analogy of turbulent dissipation and entropy production [31], the unresolved terms $\langle \Pi_{v}^{sgs}\rangle$ and $\langle \Pi_{q}^{sgs}\rangle$ can be approximated as

(11) $$\langle \Pi_{v}^{sgs}\rangle \approx \frac{\langle \bar{\rho }\rangle }{\langle \bar{T}\rangle }\langle \epsilon_{k_{sgs}}\rangle \;\mathrm{and}\;\langle \Pi_{q}^{sgs}\rangle \approx \frac{\langle \bar{\rho }\rangle \langle {\bar{c}}_{p}\rangle }{{\langle \bar{T}\rangle }^{2}}\langle \epsilon_{\theta_{sgs}}\rangle ,$$

where expressions for the dissipation rate of the subgrid-scale turbulent kinetic energy $\langle \epsilon_{k_{sgs}}\rangle$ and temperature variance $\langle \epsilon_{\theta_{sgs}}\rangle$ need to be defined. From Equation (11) it appears clearly that the physical mechanism of entropy generation in subgrid-scales is only driven by turbulent dissipation processes. Thereby, temperature fluctuations in the denominator 1/T and $1/T^{2}$ in the subgrid-scale contributions, respectively, are neglected. Applying the inertial subrange theory of isotropic turbulence (see e.g., [54,55]), it follows for the time-averaged dissipation rate of subgrid-scale kinetic energy that

(12) $$\langle \epsilon_{v,sgs}\rangle =\frac{1}{\Delta^{4}C_{S}^{4}}{\langle \nu_{sgs}\rangle }^{3},$$

where $C_{s}$ is the Smagorinsky constant and $\Delta ={\left(\Delta_{x}\Delta_{y}\Delta_{z}\right)}^{1/3}$ the grid filter width. Regarding $\langle \epsilon_{\theta ,sgs}\rangle$ the Obukhov-Corrsin inertial-convective subrange [56] scaling leads to

(13) $$\langle \epsilon_{\theta ,sgs}\rangle =\frac{4}{3C_{OC}\pi^{4/3}C_{S}^{4/3}}\frac{1}{\langle Pr\rangle }\langle \nu_{sgs}\rangle \langle \frac{\partial \bar{T}}{\partial x_{i}}\frac{\partial \bar{T}}{\partial x_{i}}\rangle ,$$

where $C_{OC}=1.34$ [55] is the coefficient of the three-dimensional temperature spectrum. Observe that in expressions Equations (12) and (13) the Smagorinsky coefficients $C_{S}$ can be related to common subgrid-scale models (e.g., $C_{W}^{2}/C_{S}^{2}=11.27$ [51] in the case of WALE) and therefore only fundamental values of model coefficients are used. A detailed derivation of Equations (12) and (13) are provided in the Appendix A and Appendix B, respectively.

It is important to mention that the relations in Equations (12) and (13) hold only true for mean values of $\langle \epsilon_{v,sgs}\rangle$ and $\langle \epsilon_{\theta ,sgs}\rangle$. However, by neglecting the fluctuation part of the subgrid-scale entropy generation, instantaneous values can be formulated as

(14) $$\Pi_{v}^{sgs}=\frac{\bar{\rho }}{\bar{T}}\frac{\nu_{sgs}^{3}}{\Delta^{4}C_{S}^{4}}\;\mathrm{and}\;\Pi_{q}^{sgs}=\frac{\bar{\rho }\;{\bar{c}}_{p}}{{\bar{T}}^{2}}\frac{4}{3C_{OC}\pi^{4/3}C_{S}^{4/3}}\frac{\nu_{sgs}}{Pr}\frac{\partial \bar{T}}{\partial x_{i}}\frac{\partial \bar{T}}{\partial x_{i}},$$

while averaging of these equations should be performed in the same manner as Equations (12) and (13).

### 2.3. Second Law-Based Performance Evaluation Criteria

Several performance evaluation criteria based on the second law analysis for thermal devices are reported in the literature (e.g., [57,58]). These criteria can be divided into performance criteria that use entropy and those that employ exergy as evaluation parameter. An overview of different performance criteria based on both, entropy and exergy, are reviewed in [57,58] and elsewhere.

In the present work, the entropy generation number *N* introduced by Bejan [14] is used to assess the overall thermodynamic performance of different jet impingement cooling designs. It represents essentially the ratio of lost exergy divided by the total exergy introduced into the system [14] and is defined in its general form as:

(15) $$N=\sum_{i}\frac{\int_{V}\langle \Pi_{i}\rangle dV}{{\dot{Q}}_{w}/T_{0}}.$$

Here $\Pi_{i}$ is the entropy generation rate due to different physical processes (*i*) (e.g., friction loss $\Pi_{v}$, heat transport $\Pi_{q}$, etc.), *V* is the volume of the thermal device, ${\dot{Q}}_{w}$ the thermal power introduced into the system and $T_{0}$ the reference ambient temperature ($T_{0}$ = 298 K). Regarding LES of thermo-fluid processes, only viscous dissipation and heat transport contribute to the generation of entropy. Thus, the entropy generation number *N* can be formulated as

(16) $$N=\underset{N_{v}}{\underbrace{\frac{\int_{V}\left(\langle \Pi_{v}\rangle +\langle \Pi_{v}^{sgs}\rangle \right)dV}{{\dot{Q}}_{w}/T_{0}}}}+\underset{N_{q}}{\underbrace{\frac{\int_{V}\left(\langle \Pi_{q}\rangle +\langle \Pi_{q}^{sgs}\rangle \right)dV}{{\dot{Q}}_{w}/T_{0}}}},$$

where $N_{v}$ is the exergy loss related to viscous dissipation and $N_{q}$ to heat transport.

## 3. Configuration

The LES framework for entropy generation analysis as introduced above is applied in this work to evaluate and optimize the thermodynamic performance of a turbulent square jet impinging on a heated solid surface. The heat and fluid flow within this turbulent impinging jet was investigated experimentally and numerically by the authors in previous studies using particle image velocimetry (PIV) and DNS technique, respectively (see [9,49]). The configuration under consideration is therefore only shortly introduced here. Further information on the test case and a detailed description of the general flow features, heat transport and turbulence characteristics can be found in the previous studies [9,49].

**Figure 1:**
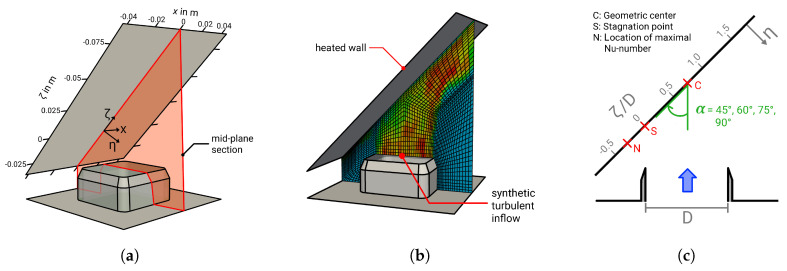
Computational domain (**a**), slice through the numerical grid at mid-plane section (**b**), and description of the coordinate system and inclination angle α (**c**) of the impinging cooling configuration. C: geometric center point of the jet; S: stagnation point; N: location of maximal Nusselt number.

A schematic of the configuration investigated in the LES study is provided in Figure 1, where (a) shows the three-dimensional computational domain, (b) a slice through the numerical grid at mid-plane section, and (c) a description of the coordinate system and the inclination angle α. Notice that an additional coordinate system is introduced with η representing the wall-normal direction and ζ denoting the direction along the wall. The origin of the introduced coordinate system (ζ,η) is located at the stagnation point *S* of the jet (see Figure 1c). Thus, it is located directly at the geometric origin *C* of the jet in the case of an inclination of α=90° and shifted approximately half a diameter away from the geometric origin *C* towards the compression side in the case of α=45° (see also [9,49]). The point *N* in Figure 1c represents the location where the Nusselt number is maximal.

In line with the reference experiment and DNS (see [9,49]), a turbulent jet of dry air ($T_{inlet}$ = 290 K, *p* = 1.01325 bar) leaves a square nozzle (*D* = 40 mm) and impinges on a heated flat wall, which has a constant wall temperature of $T_{wall}$ = 330 K and a jet-to-plate spacing of H/D = 1. At the impinged wall, the jet is divided into two opposed wall-jets directed outward along the solid wall and gets heated up. In contrast to the experiment and DNS study in which only an inclination angle of α = 45° and a Reynolds number of Re = 5000 is examined, further inclination angles (α = 45°, 60°, 75°, 85°, 86°, 87°, 88°, 89°, 90°) and Reynolds numbers (Re = 5000, 10,000, 20,000) are considered in the LES study. Important dimensions and operating conditions of the configurations investigated in the LES study are summarized in Table 1.

Regarding the boundary conditions of the LES study, synthetic turbulent inflow conditions are employed at the nozzle exit section. Thereby, the axial velocity is set to the bulk velocity (e.g., $U_{inlet}$ = 1.949 m/s for Re = 5000) and artificial turbulence producing spatially and temporally correlated velocity fluctuations are superimposed based on the digital filtering approach proposed by Klein et al. [59]. The velocity fluctuations are approximated to be isotropic with a turbulence intensity of $I_{inlet}$ = 9.5% similar to the experiment (see [49]). The temperature at the inflow is set to a constant value of $T_{inlet}$ = 290 K. At the outlets, a velocity inlet/outlet boundary condition is used to allow entrainment of air from the surrounding. Thereby, the incoming fluid velocity is obtained by the internal cell value, while a zero Neumann condition is applied in the case of outflow. For the temperature, the zero Neumann condition is set at the outlets. At the walls, the no-slip condition is used for the velocity, and the zero Neumann condition is applied for the temperature, except at the impinged wall, where the temperature is set to a constant value of *T* = $T_{wall}$ = 330 K.

A summary of the parametric study is given in Table 2. Thereby, block-structured, three-dimensional numerical grids are used for the LES simulations. The numerical grids are refined towards the impinged wall to ensure that the near-wall region is fully resolved in the LES. To determine the grid sensitivity of the results, a systematic grid variation study is performed for each Reynolds number for an inclination angle of 45° (see Table 1, cases: 1–3, 11–13, 22–23). Results of the grid sensitivity study for Re = 5000 (Table 1, cases 1–3) are provided in Section 4.1. Similar results are obtained for the higher Reynolds numbers and therefore not shown here.

## 4. Results and Entropy Generation Analysis

To establish the validity of the simulations and to analyze the grid sensitivity, LES predictions of the impingement jet with Re=5000 and α=45° are first compared with DNS data from the literature (see [9,49]). Notice that impressive agreements have been reported between the DNS results and experimental data for the flow field in the previous study [49]. Then, after establishing the validity of the LES, general heat and fluid flow features of the inclined impinging jet are analyzed with respect to different inclination angles and jet Reynolds numbers. Finally, the entropy generation in the impinging jet configurations is analyzed and the thermodynamic efficiency of the different arrangements are evaluated based on the method of entropy generation minimization using the entropy generation number [14].

### 4.1. Comparison with DNS and Grid Sensitivity

**Figure 2:**
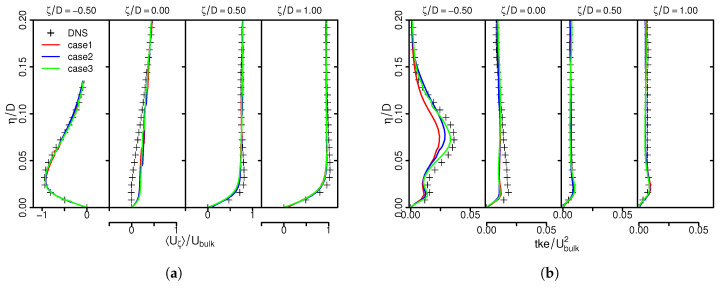
Mean wall-normal velocity (**a**) and turbulent kinetic energy tke (**b**). Comparison of LES results and DNS data.

Figure 2 shows the predicted LES mean velocity component in wall-parallel direction $\langle U_{\zeta }\rangle$ (a) and turbulent kinetic energy tke (b) in comparison with DNS data as a function of the wall-normal direction η/D. All results are normalized by the jet’s bulk velocity $U_{bulk}$.

As it can be observed in Figure 2a, predicted mean velocities agree well with the reference DNS with minor discrepancies at the stagnation region. Slight deviation from DNS for tke predictions can be observed, especially at the stagnation region (see Figure 2b). However, characteristic flow features of the 45°-inclined impinging jet are well reproduced by the LES. This holds true for all numerical grids under consideration and confirms that the numerical setup and spatial resolution are appropriate to describe the turbulent flow field of this configuration. A similar conclusion can be drawn for predicted mean and rms temperature profiles. They are therefore not shown here.

**Figure 3:**
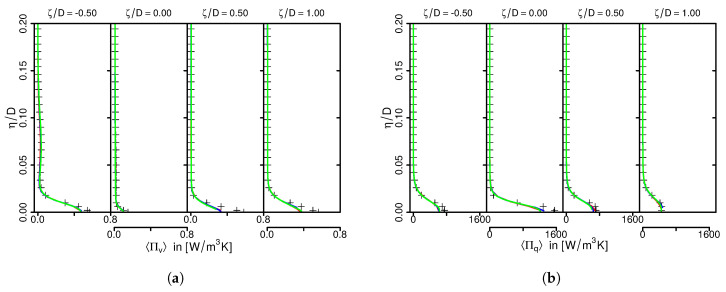
Time-averaged entropy production rates by viscous dissipation (**a**) and heat transport (**b**). Comparison of LES predictions and DNS data. Legend see Figure 2.

Finally, predicted time-averaged entropy production rates by viscous dissipation $\langle \Pi_{v}\rangle$ and heat transport $\langle \Pi_{q}\rangle$ are compared with the generated DNS dataset in Figure 3 for different spatial resolutions.

Entropy production is high around the wall and decreases rapidly away from it. Thereby, entropy is predominantly generated by heat transport rather than viscous dissipation, in particular at the stagnation region. This behavior is well reproduced by the LES, even for the coarsest grid. Furthermore, LES predictions are very close to the DNS, which establishes the predictive capability of the present LES framework in terms of entropy production. It can be therefore used for further investigations of different inclination angles and Reynolds numbers in the inclined impinging jet configuration.

### 4.2. Influence of Reynolds Number and Plate Inclination

**Figure 4:**
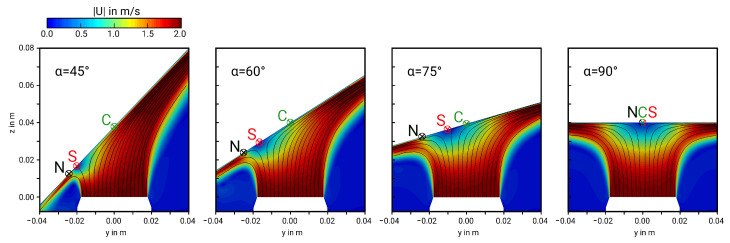
Mean velocity field at mid-plane section of the impinging jet for different inclination angles α. *S*: stagnation point; *N*: location of maximal Nu-number; *C*: geometric center of the jet (for Re=5000).

After establishing the validity of the LES, the influence of the inclination angle and Reynolds number on thermal and fluid flow properties is analyzed now. For this purpose, Figure 4 shows the time-averaged velocity magnitude field at mid-plane section of the jet. Black solid lines represent streamlines of the mean flow field, *S* the stagnation point, *N* the location of maximum Nusselt number and *C* the geometric center of the jet. Results are exemplarily shown for Re = 5000 and α = 45°, 60°, 75°, 90° (cases 3, 4, 5, 10 in Table 2).

**Figure 5:**
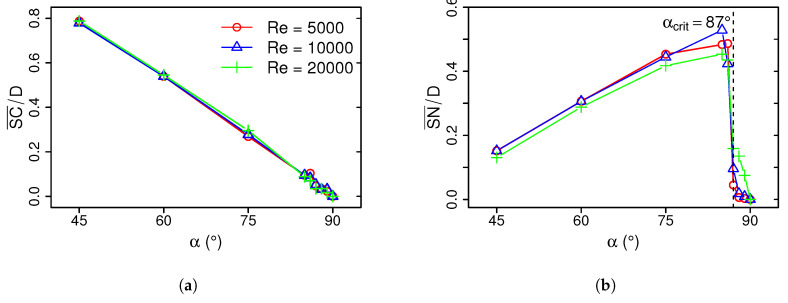
Displacement distance between stagnation point and (**a**) geometric center of the jet $\bar{SC}$, and (**b**) location of maximal Nusselt number $\bar{SN}$ for different inclination angles and Reynolds numbers.

It can be clearly seen in Figure 4 that the stagnation point *S* becomes shifted towards the compression side with decreasing inclination angle α. In this respect, it is interesting to observe that, in contrast to an inclination angle of α=90°, the location of maximum Nusselt number *N* and the stagnation point *S* do not coincide in the case of α<90°. Instead, the location of *N* is slightly shifted towards the compression side of the jet, in between the stagnation point and the opposed wall-jet region. Here, the direction of the flow changes suddenly and the fluid is subject to a strong acceleration. As pointed out in [9], turbulent kinetic energy is very high at this region and turbulence-induced mixing among other thermo-fluid processes enhance the heat transfer, which leads to very high local Nusselt numbers at this specific region. With increasing α, the acceleration of the fluid along with the turbulence-induced mixing at the wall-jet region decrease and consequently also the local Nusselt numbers. Once a critical inclination angle of $\alpha \geq \alpha_{crit}$ is reached, heat transfer at the stagnation point exceeds that at the wall-jet region and *S* and *N* coincide at the same position. These anomalies of the stagnation point and peak Nusselt number are quantified in Figure 5, which depicts the displacement distance between the stagnation point and: (a) the geometric center of the jet $\bar{SC}$, and (b) the location of maximal Nusselt number $\bar{SN}$ as a function of α for Re=5000, 10,000, and 20,000.

As expected, $\bar{SC}$ decreases linearly with increasing inclination angle and is zero for α=90° (see Figure 5a). In contrast, the displacement $\bar{SN}$ is maximal for $\alpha_{crit}=86{}^{\circ}$ and decreases with decreasing inclination angle (see Figure 5b). Therefore, small deviations from α=90° results in large values of $\bar{SN}$. For α=90°, the maximal Nusselt number occurs exactly at the stagnation point. This characteristic behavior of $\bar{SC}$ and $\bar{SN}$ is independent of the Reynolds number and predominantly caused by geometric constraints rather than turbulence dynamics.

**Figure 6:**
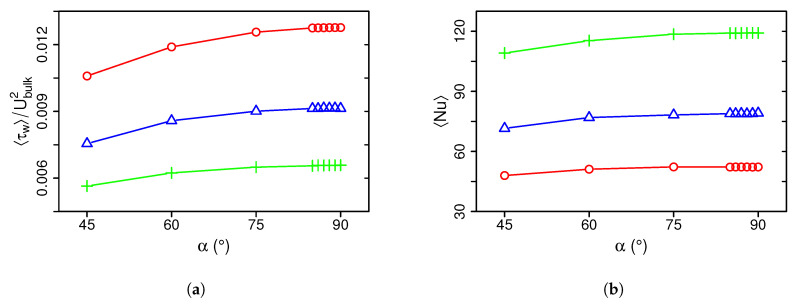
Mean wall shear stresses (**a**) and Nusselt numbers (**b**) at the impinged wall for different inclination angles and Reynolds numbers. Legend see Figure 5.

To quantify the flow/wall interaction and the heat transfer performance of the impingement cooling device, Figure 6 shows mean wall shear stresses $\langle \tau_{w}\rangle$ (a) and mean Nusselt numbers $\langle Nu\rangle$ (b) for different inclination angles and Reynolds numbers. Values of $\langle \tau_{w}\rangle$ are normalized by the bulk velocity of the jet.

The mean wall shear stress $\langle \tau_{w}\rangle$ averaged over the impinged wall is highest in the 90°-configuration and decreases with decreasing inclination angle (see Figure 6a). Similar to the skin friction coefficient in pipe or channel flows, non-dimensional values of $\langle \tau_{w}\rangle$ decrease with increasing Reynolds number, which holds true for mean and maximal values of $\langle \tau_{w}\rangle$. Thereby, non-dimensional values of $\langle \tau_{w}\rangle$ are approximately five times higher in the impinging jet configuration than skin friction coefficients found in pipe or channel flows with similar Reynolds numbers (for comparison see [60]) reflecting the strong flow/wall interaction in the impinging jet configuration. In analogy to wall shear stresses, the mean Nusselt number averaged over the impinged wall decreases with decreasing inclination angle α and Reynolds number (see Figure 6b). By using the empirical correlation of Dittus and Boetler for forced convection in turbulent pipe flow [61], it appears that the impinging jet configuration enables approximately three times higher Nusselt numbers for the same Reynolds numbers. This establishes the high efficiency of jet impingement cooling arrangements to transfer thermal energy between a heated surface and a coolant fluid.

### 4.3. Entropy Generation Analysis and Optimal Design

As an effective cooling device, the impingement cooling arrangement should provide a high heat transfer performance and small friction loss to use the available energy efficiently. However, the heat transfer enhancement in thermal devices is usually associated with an increase of friction loss, which is also the case in jet impingement cooling arrangements as determined before (compare Figure 5a,b). Therefore, to find an optimal trade-off between heat transfer enhancement and friction loss by selecting the best flow conditions and impingement configuration is a challenging task. In this respect, the significance of both heat transfer enhancement and friction loss, can be evaluated by means of entropy generation of the respective impingement setup with smallest entropy generation in the thermodynamic optimal design.

**Figure 7:**
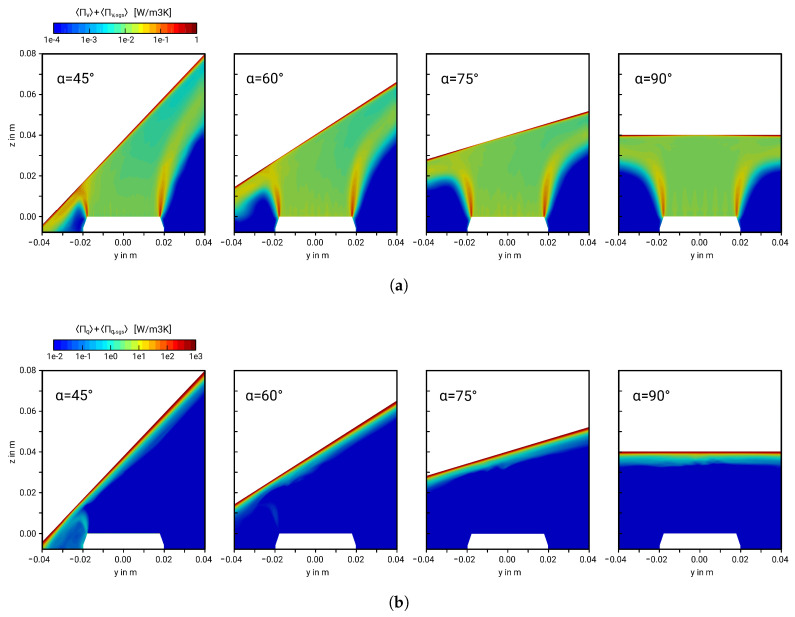
Predicted entropy generation maps related to viscous dissipation (**a**) and heat transport (**b**). Results are shown for the sum of resolved entropy production rates and subgrid contribution.

For a proper evaluation of the different impingement setups, the external wall heat flux at the impinged wall and the heat transfer area should be fixed in the entropy generation analysis to obtain the general performance of the system independent of the boundary conditions. Instead of a fixed wall temperature, a constant heat flux of ${\dot{q}}_{w}$ = 1000 W/m${}^{2}$ is therefore used in the present performance evaluation study for all cases. This value is selected based on the averaged heat flux obtained in the DNS study with α=45° (see [9]). The resulting time-averaged entropy generation maps related to friction (a) and heat transport (b) are depicted in Figure 7 for Re=5000.

Regarding friction losses (see Figure 7a), entropy is primarily produced at the shear layer of the jet and in the vicinity of the impinged wall due to steep velocity gradients. Furthermore, entropy generation rates related to viscous dissipation are also high at the opposed wall-jet region on the compression side of the impinging jet. This can be attributed to the sudden change in the flow direction and the strong acceleration of the fluid in wall-parallel direction in this region, which lead to intense shearing. In contrast, entropy generation related to heat transport is predominantly concentrated at the near-wall region (see Figure 7b), where temperature gradients are high. Thereby, by comparing Figure 7a,b, it appears that entropy is predominantly generated by heat transport rather than viscous dissipation for the present impingement cooling setup.

**Figure 8:**
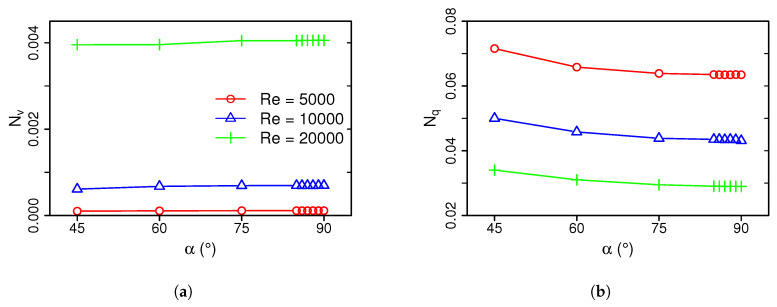
Entropy generation numbers of friction loss (**a**) and heat transport (**b**) as a function of inclination angle α for Re = 5000, 10,000, 20,000.

Once the main sources of irreversibilities evolving in the impingement cooling arrangements have been identified, the amount of exergy loss is quantified next. For this purpose, Figure 8 shows the entropy generation numbers of friction loss $N_{v}$ and heat transport $N_{q}$ of the impingement cooling device with respect to the inclination angle and Reynolds number (see Equation (16)).

As it can be seen in Figure 8a, friction losses increase slightly with increasing inclination angle α and are highest in the 90°-configuration. Friction losses are more significant for higher Reynolds number, which is approximately 40 times higher for Re = 20,000 than in the case of Re=5000. Nevertheless, the exergy loss caused by viscous dissipation appears relatively small compared to the loss by heat transport, which is about one order of magnitude larger for the selected operating conditions (see Figure 8b). Thereby, a minimum in $N_{q}$ can be observed for the 90°-configuration, which is attributed to the higher heat transfer coefficients of this configuration (see Figure 6b). Furthermore, increasing the Reynolds number enhances the heat transfer considerably. Therefore, it can be concluded that small inclination angles α are desirable in terms of friction losses, while a 90°-configuration is most effective in terms of heat transfer. Moreover, an increase in Reynolds number not only intensifies the heat transfer, but also increases friction losses.

**Figure 9:**
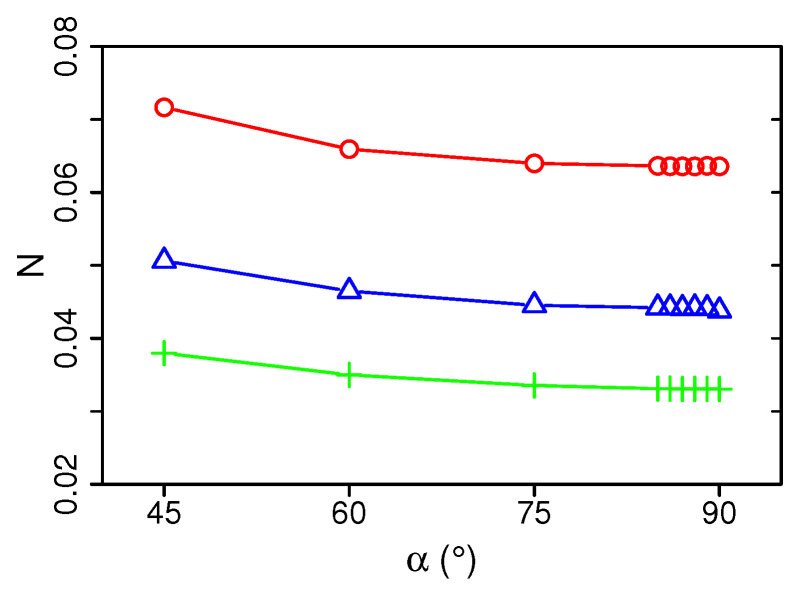
Total entropy generation number *N* as a function of inclination angle α for Re=5000, 10,000, 20,000. Legend see Figure 8.

Finally, the thermodynamic efficiency of the different impingement setups is determined by using the total entropy generation number *N* (see Equation (16)). Figure 9 depicts the total exergy loss of the impingement cooling devices as a function of inclination angle and Reynolds number.

For the present operating conditions, Figure 9 clearly shows that the α=90° configuration is the thermodynamically optimal setup with the lowest value of *N* of all configurations under consideration. Therefore, it allows the most efficient use of energy in the impingement cooling device. In this respect, it is further observed that the overall efficiency can be significantly improved by increasing the Reynolds number from Re=5000 to Re = 20,000. However, the analysis of the frictional exergy losses in Figure 9 suggests that there is still scope for further improvement at higher Reynolds numbers.

## 5. Conclusions

In this paper, novel model equations that allow calculation of entropy generation rates in the post-processing phase of LES for turbulent heat and fluid flows have been suggested. The resulting LES approach was used to perform second law analysis of a turbulent jet impinging on a heated surface for the first time in the LES framework to point out the influences of impingement angle and Reynolds number on the thermodynamic performance. Some important observations from this LES study can be outlined as follows:

(1) It appears that the location of the stagnation point and that of the maximal Nusselt number differ in the case of plate inclination, except for the case with α=90°. Both are shifted towards the compression side of the jet with decreasing inclination. Only in the case of α=90°, the stagnation point and the location of maximal heat transfer coincide at the same point.
(2) Wall shear stresses and Nusselt numbers averaged over the impinged wall are high in the 90°-configuration and decreases with decreasing inclination angle α.
(3) When dealing with entropy generation analysis in the LES framework, it turned out that entropy generation apart from solid walls is predominantly a subgrid-scale process and therefore accurate closure approaches are of profound importance. The formulations based on inertial-convective range scaling as suggested in Section 2 proved to be a promising approach for entropy generation analysis in LES as testified by comparison with DNS data (see Section 4.1 and Appendix C).
(4) Regarding the optimal design of impingement cooling devices, this LES study suggests that an inclination angle of 90° allows the most efficient use of energy. In addition, increasing the Reynolds number intensifies the heat transfer and increases the second law efficiency of the system.

## Figures and Tables

**Table 1:** Important dimensions and operating conditions of the jet impingement cooling configuration.

Property	Description	Value
α	inclination angle of the plate	45°, 60°, 75°, 85°, 86°, 87°, 88°, 89°, 90°
*D*	nozzle exit diameter	40 mm
*H*	jet-to-plate distance	40 mm
$T_{inlet}$	temperature at the nozzle exit	290 K
$T_{wall}$	temperature of the heated wall	330 K
*p*	ambient pressure	1.01325 bar
Re	Reynolds number based on the nozzle exit diameter	5000, 10,000, 20,000
Pr	molecular Prandtl number	0.71
$I_{inlet}$	turbulence intensity at the inflow	9.5%

**Table 2:** Summary of the parametric study for the entropy production analysis in the impingement cooling device. α: inclination angle; Re: jet Reynolds number.

Case	α	Re	No. of Cells
1, 2, 3	45°	5000	1.0 million, 1.7 million, 3.1 million
4	60°	5000	1.7 million
5	75°	5000	1.7 million
6	85°	5000	1.7 million
7	86°	5000	1.7 million
8	88°	5000	1.7 million
9	89°	5000	1.7 million
10	90°	5000	1.7 million
11, 12, 13	45°	10,000	1.7 million, 3.0 million, 4.8 million
14	60°	10,000	3.0 million
15	75°	10,000	3.0 million
16	85°	10,000	3.0 million
17	86°	10,000	3.0 million
18	87°	10,000	3.0 million
19	88°	10,000	3.0 million
20	89°	10,000	3.0 million
21	90°	10,000	3.0 million
22, 23, 24	45°	20,000	3.0 million, 4.8 million, 7.6 million
25	60°	20,000	4.8 million
26	75°	20,000	4.8 million
27	85°	20,000	4.8 million
28	86°	20,000	4.8 million
29	87°	20,000	4.8 million
30	88°	20,000	4.8 million
31	89°	20,000	4.8 million
32	90°	20,000	4.8 million

**Figure A1:**
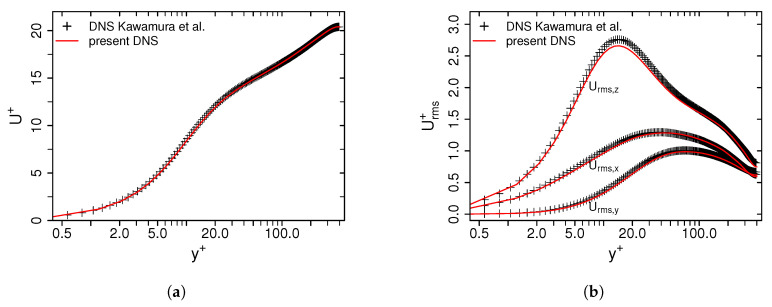
Mean (**a**) and rms (**b**) velocities as a function of the non-dimensional wall distance $y^{+}$. Comparison of results from the present DNS with the reference DNS dataset of Kawamura et al. [62].

**Figure A2:**
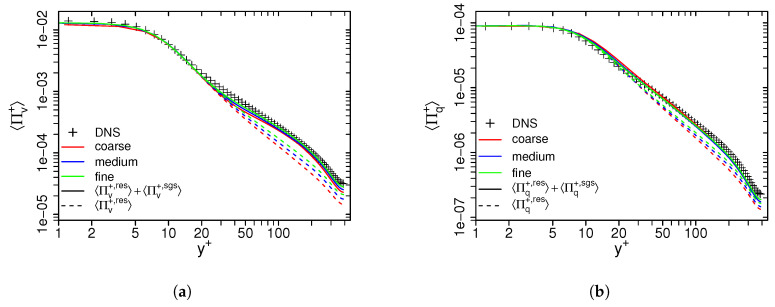
Time-averaged entropy production rates by viscous dissipation (**a**) and heat transport (**b**). Comparison of LES predictions with DNS results.

**Figure A3:**
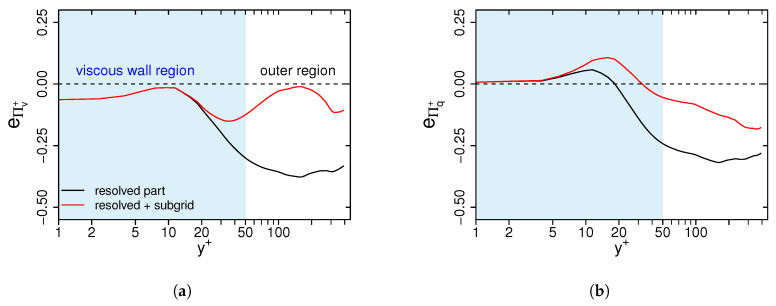
Relative error of LES predictions of resolved and total entropy production rates by viscous dissipation (**a**) and heat transport (**b**). Results are evaluated for the LES grid with 2 million control volumes.

## Abbreviations

The following abbreviations are used in this manuscript:

CFD	computational fluid dynamics
DNS	direct numerical simulation
FDF	filtered density function
LES	large eddy simulation
PIV	particle image velocimetry
RANS	Reynolds-averaged Navier-Stokes
sgs	subgrid-scale
tke	turbulent kinetic energy
WALE	wall-adapting linear eddy viscosity model

## Appendix A. Determination of the Dissipation Rate of Subgrid-Scale Kinetic Energy

Assuming high-Reynolds-number homogeneous isotropic turbulence and the existence of an intermediate range of scales, the three-dimensional spectrum of kinetic energy integrated over all wavenumbers of magnitude κ follows the well-known universal form of

(A1) $$E\left(\kappa \right)=C_{K}{\langle \epsilon_{v}\rangle }^{2/3}\kappa^{-5/3},$$

where $C_{K}=1.5$ is the Kolmogorov constant and $\langle \epsilon_{v}\rangle$ is the dissipation rate of turbulent kinetic energy. From this, the kinetic energy of residual motion separated by the cut-off wavenumber $\kappa_{c}=\pi /\Delta$ is

(A2) $$\langle k_{sgs}\rangle =\int_{\pi /\Delta }^{\infty }E\left(\kappa \right)d\kappa =\frac{3}{2}C_{K}{\langle \epsilon_{v}\rangle }^{2/3}{\left(\frac{\pi }{\Delta }\right)}^{-2/3},$$

where Δ is the grid filter width defined here as $\Delta ={\left(\Delta_{x}\Delta_{y}\Delta_{z}\right)}^{1/3}$. By means of the relations $\langle k_{sgs}\rangle ={\langle \nu_{sgs}\rangle }^{2}/\left(\Delta C_{v}\right)$, $C_{v}=C_{s}^{4/3}C_{\epsilon ,v}^{1/3}$, $C_{\epsilon_{v}}=\pi {\left(\frac{3}{2}C_{K}\right)}^{-3/2}$ (see [54,55]) and assuming that $\langle \epsilon_{v,sgs}\rangle \approx \langle \epsilon_{v}\rangle$, it follows for the dissipation rate of subgrid-scale kinetic energy that

(A3) $$\begin{array}{cc} \langle \epsilon_{v,sgs}\rangle & ={\left(\frac{2}{3C_{K}}\right)}^{3/2}\frac{\pi }{\Delta }{\langle k_{sgs}\rangle }^{3/2}\\ & =\frac{1}{\Delta^{4}C_{s}^{4}}{\langle \nu_{sgs}\rangle }^{3}, \end{array}$$

where $C_{s}$ is the Smagorinsky constant. The first formulation in Equation (A3) can be applied in the case of first order modeling approaches with one-equation models while the second formulation is suitable for first order modeling with zero-equation models.

## Appendix B. Determination of the Dissipation Rate of Subgrid-Scale Temperature Variance

In accordance to the kinetic energy spectrum in the inertial subrange, the temperature variance spectrum within the inertial-convective subrange reads

(A4) $$E_{\theta }\left(\kappa \right)=C_{OC}\langle \epsilon_{\theta }\rangle {\langle \epsilon_{v}\rangle }^{-1/3}\kappa^{-5/3},$$

where $C_{OC}=1.34$ [55] is Batchelor’s coefficient and $\langle \epsilon_{\theta }\rangle$ the dissipation rate of temperature variance. From this, the temperature variance of residual motions separated by the cut-off wavenumber ${}_{\theta }\kappa_{c}=\kappa_{c}Pr^{3/4}=\pi /\Delta Pr^{3/4}$ [56] is

(A5) $$\langle \theta_{sgs}\rangle =\int_{\pi /\Delta Pr^{3/4}}^{\infty }E_{\theta }\left(\kappa \right)d\kappa =\frac{3}{2}C_{OC}\langle \epsilon_{\theta }\rangle {\langle \epsilon_{v}\rangle }^{-1/3}{\left(\frac{\pi }{\Delta }\right)}^{-2/3}{\langle Pr\rangle }^{-1/2}.$$

Analogously, the mean square of filtered gradient of temperature can be obtained as

(A6) $$\langle \frac{\partial \bar{T}}{\partial x_{i}}\frac{\partial \bar{T}}{\partial x_{i}}\rangle =\int_{0}^{\pi /\Delta Pr^{3/4}}\kappa^{2}E_{\theta }\left(\kappa \right)d\kappa =\frac{3}{4}C_{OC}\langle \epsilon_{\theta }\rangle {\langle \epsilon_{v}\rangle }^{-1/3}{\left(\frac{\pi }{\Delta }\right)}^{4/3}\langle Pr\rangle .$$

Dividing Equations (A5) and (A6) side by side leads to the following expression for the subgrid-scale temperature variance

(A7) $$\langle \theta_{sgs}\rangle =\frac{2}{\pi^{2}}{\langle Pr\rangle }^{-3/4}\Delta^{2}\langle \frac{\partial \bar{T}}{\partial x_{i}}\frac{\partial \bar{T}}{\partial x_{i}}\rangle .$$

Finally, from Equations (A3), (A5) and (A7), the dissipation rate of subgrid-scale temperature variance is given as

(A8) $$\begin{array}{cc} \langle \epsilon_{\theta ,sgs}\rangle & =\frac{2\pi }{3C_{OC}}{\left(\frac{2}{3C_{K}}\right)}^{1/2}\frac{\langle \theta_{sgs}\rangle {\langle k_{sgs}\rangle }^{1/2}}{\Delta }{\langle Pr\rangle }^{1/2}\\ & =\frac{4}{3C_{OC}\pi^{4/3}C_{s}^{4/3}}\langle \nu_{sgs}\rangle \langle \frac{\partial \bar{T}}{\partial x_{i}}\frac{\partial \bar{T}}{\partial x_{i}}\rangle {\langle Pr\rangle }^{-1}. \end{array}$$

Thereby, the first formulation in Equation (A8) can be applied in the case of first order modeling approaches with one-equation models while the second formulation is suitable for first order modeling with zero-equation models.

## Appendix C. Evaluation of the Subgrid-Scale Modeling of Entropy Generation

To evaluate the proposed modeling approach of subgrid-scale entropy generation rates, LES predictions of entropy generation rates in a turbulent heated channel flow are compared with DNS results. In this respect, it is worth mentioning that reference experimental or DNS data regarding entropy production rates in turbulent heated near-wall flows are to the authors knowledge not available in the literature. Therefore, a DNS of a turbulent heated channel flow at $Re_{\tau }=395$ and Pr=0.71 has been performed to generate a reliable DNS database of entropy production rates for such flow configurations. The numerical setup is the same as that of the DNS of Kawamura et al. [62]. The numerical grid of the DNS consists of approximately 10 million control volumes and is refined in the near-wall region to ensure a non-dimensional wall distance smaller than one. The ratio of Kolmogorov length scale and mean grid width $\eta_{K}/\Delta$ is smaller than 2.5 in the entire domain, which confirms appropriate spatial resolution. A comparison of mean and rms velocities from the present DNS with the DNS dataset of Kawamura et al. [62] is shown in Figure A1.

As it can be clearly seen in Figure A1, mean and rms profiles of the velocity agree very well with the reference DNS data, which establishes the validity of the present DNS. Similar results are obtained for mean and rms temperatures and for the dissipation rates of turbulent kinetic energy and temperature variance. These results are therefore not shown here.

In the LES evaluation study, three numerical grids with 0.6, 1.1 and 2 million control volumes are applied that are refined in the near-wall region to ensure a non-dimensional wall distance smaller than one. Regarding subgrid-scale modeling of momentum and heat transport, the WALE model [51] with a standard model coefficient of $C_{W}=0.5$ and the linear eddy diffusivity model with $Pr_{sgs}=1$ are employed to close the LES balance equations (see Section 2).

Figure A2 shows LES predictions of time-averaged entropy production rates by viscous dissipation $\langle \Pi_{v}\rangle$ and by heat transport $\langle \Pi_{q}\rangle$ in comparison with the generated DNS dataset. Thereby, $\langle \Pi_{v}\rangle$ is normalized by $T_{\tau }\nu /\left(\rho u_{\tau }^{4}\right)$ and $\langle \Pi_{q}\rangle$ by $2\nu T_{\tau }^{2}\lambda /\left(\rho c_{p}Pr\right)$, where $T_{\tau }=q_{w}/\left(\rho c_{p}u_{\tau }\right)$ is the friction temperature and $q_{w}$ the heat flux from the walls. Dashed lines in Figure A2 denote the resolved entropy production rates by the LES ($\langle \Pi_{v}^{res}\rangle$ and $\langle \Pi_{q}^{res}\rangle$), while solid lines represent the sum of the resolved part and the subgrid-scale contribution. Notice that the results are presented in a double logarithmic scaled graph to visualize the large range of entropy production rates.

Entropy generation rates are high at the viscous sublayer ($y^{+}<5$), decrease rapidly in the buffer layer ($y^{+}<30$) and they are low at the log-law region ($y^{+}>30$). This characteristic physical behavior of entropy generation in wall-bounded heated flows is well retrieved by the LES approach. Regarding the modeling of the subgrid-scale entropy production rates, it can be clearly seen that predictions of $\langle \Pi_{v}\rangle$ and $\langle \Pi_{q}\rangle$ including the subgrid-scale contribution are more reliable than taking only the resolved part. Furthermore, the residual part is small in the near-wall region and increases significantly away from the wall. This seems reasonable since the near-wall region is fully resolved in the LES simulations, thus subgrid-scale quantities vanish towards the wall. In the outer region ($y^{+}>40$) subgrid-scale entropy production rates are high, approximately of the same order as the resolved part. In this region, total entropy production rates are slightly underestimated by the LES modeling approach, even when the residual contribution is added. Nevertheless, the overall agreement of LES predictions with the DNS data is quite good considering the large contribution of the subgrid-scale modeling.

Subsequently, the prediction accuracy of entropy production rates with and without adding the residual contribution is quantified by means of an error analysis. For this purpose, the relative error is calculated for different wall distances as

(A9) $$e_{\Pi_{i}}=\frac{\langle \Pi_{i}^{DNS}\rangle -\langle \Pi_{i}^{LES}\rangle }{\langle \Pi_{i}^{DNS}\rangle }.$$

Results of the error analysis are depicted in Figure A3 for the LES grid with 2 million control volumes. Here, black lines represent the error calculated for entropy production rates without adding the residual contribution and red lines the relative error of entropy production rates including the subgrid-scale contribution.

Since the near-wall region is fully resolved in the LES study, the contribution from the subgrid-scale modeling of entropy generation rates is small at the viscous wall region and, therefore, relative prediction errors are small as well in this region. Apparent in Figure A3, the subgrid-scale contributions of entropy production increase further away from the wall. Thereby, relative prediction errors increase due to the additional error contribution from the subgrid-scale modeling. However, it can be clearly seen that adding the modeled subgrid-scale contribution significantly improves the prediction accuracy of entropy production rates. This holds true for both, $e_{\Pi_{v}^{+}}$ and $e_{\Pi_{q}^{+}}$, and confirms the reliability of the present modeling approach.
